# Time-Dependent Toxicities of Quorum Sensing Inhibitors to *Aliivibrio fischeri* and *Bacillus subtilis*

**DOI:** 10.1177/1559325818822938

**Published:** 2019-02-25

**Authors:** Yueheng Zhang, Jinyuan Song, Ting Wang, Haoyu Sun, Zhifen Lin, Yinjiang Zhang

**Affiliations:** 1 State Key Laboratory of Pollution Control and Resource Reuse, College of Environmental Science and Engineering, Tongji University, Shanghai, China; 2 Solid Waste and Chemicals Management Center, Ministry of Environmental Protection, Beijing, China; 3 Shanghai Institute of Pollution Control and Ecological Security, Shanghai, China; 4 Shanghai Key Lab of Chemical Assessment and Sustainability, Shanghai, China; 5 State Key Laboratory of Environmental Chemistry and Ecotoxicology, Research Center for Eco-Environmental Sciences, Chinese Academy of Sciences, Beijing, China; 6 College of Marine Ecology and Environment, Shanghai Ocean University, Shanghai, China

**Keywords:** quorum sensing inhibitor, hormesis, *Bacillus subtilis*, *Aliivibrio fischeri*, risk assessment

## Abstract

Quorum sensing inhibitors (QSIs) are being used widely as a promising alternative to antibiotics and drawing attention as potential pollutants. However, the assessment methods of the toxicities of QSIs, including model organism and affecting time, have not been established. To investigate how model organism and acting time impact the toxicities of QSIs, the effect of 4 QSIs to *Aliivibrio fischeri* and *Bacillus subtilis* were determined at different exposing time in the present study. The results showed that the toxic effects of QSIs to gram-negative bacteria (*A fischeri*) and gram-positive bacteria (*B subtilis*) were different and time dependent. As for *A fischeri*, QSI (furaneol acetate, FA) merely showed inhibition on the bioluminescence from hours 1 to 2. But from hours 3 to 6, low concentration FA exerted stimulation on the bioluminescence. Then, this stimulation disappeared from hours 7 to 14, and after hour 15 the stimulation appeared again. That is to say, QSIs showed intermittent hormesis effect on the bioluminescence of *A fischeri*. By contrast, only inhibition was observed in the toxicity test process of QSIs to *B subtilis*. As exposing time goes, the inhibition weakened gradually when FA was at low concentration regions. What is more, in the present, study toxic mechanisms were also discussed based on model organisms and exposing time. This study demonstrates appreciable impacts of model organism and exposing time on toxicities of QSIs and provides a theoretical basis for risk assessments after QSIs being widely used into the environment.

## Introduction

Quorum sensing inhibitors (QSIs) are a new class of antibiotic drugs that act on the quorum sensing (QS) system of bacteria. As QSIs are unlikely to make bacteria to generate antibiotic resistance,^1^ they are regarded as promising antibiotic agents instead of antibiotics against the abuse of antibiotics and the dissemination of resistance genes.^2,3^ Then, whether the spread of QSIs will bring about negative impacts on the environment and what impact they will cause are important questions worthy of note.

A considerable number of studies have addressed the biological effect of synthetic QSIs to microorganisms^4–10^ and revealed the effect of QSIs on bacterial biofilm formation, bioluminescence, virulence production, and growth. For instance, Hentzer et al demonstrated that furanone can inhibit virulence factor expression and increase bacterial susceptibility to tobramycin.^11^ However, these studies are far from perfect due to the fact that they mainly focused on gram-negative bacteria as a model organism and the toxicity tests were operated at a certain exposing time. In the natural environment, there exist gram-positive bacteria as well, and the acting time of pollutants may cover each stage of bacterial growth. Therefore, it is worthwhile to investigate how model organism and exposing time impact the toxicities of QSIs to microorganism.

**Figure 1.**
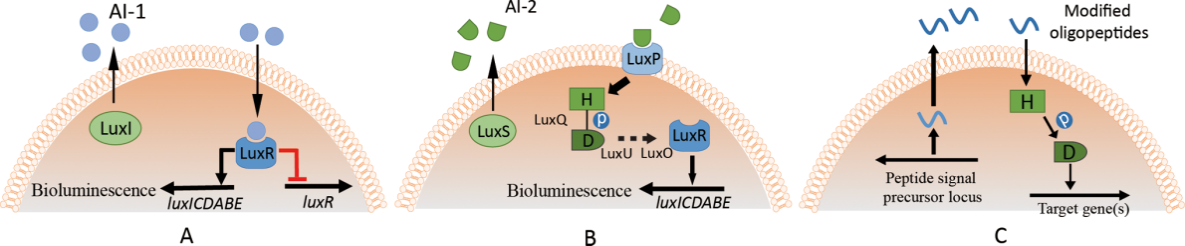
Three canonical quorum sensing systems. (A) LuxI system is in charge of intraspecific communications of gram-negative bacteria. N-acyl homoserine lactones (AHLs) are the signal molecules, which is called AI-1; (B) LuxS system is for interspecific communications among both gram-negative and gram-positive bacteria, using derivatives of 4,5-dihydroxy-2,3-pentanedione (DPD) as the signal molecules, also known as AI-2; (C) the system that is regulated by oligopeptide compounds for intraspecific communication in gram-positive bacteria (data from^17^).

It is well known that the toxic mechanism of QSIs is to block QS pathways by competing with the signaling molecules to bind to receptor proteins, as QSIs are the analogs of signal molecules of QS.^12^ Quorum sensing refers to a cell–cell communication that controls the gene expression involving physiological functions,^13^ such as bioluminescence,^14^ biofilm formation,^15^ and the production of toxin factors.^16^ According to chemical structures of signal molecules and pathways, QS system can be mainly divided into 3 categories. Figure 1 shows 3 canonical QS systems.

*Aliivibrio fischeri* is gram-negative bacteria that has a well-studied QS, namely, LuxI/AI-1 and LuxS/AI-2. LuxI/AI-1 includes 2 signal molecules, C6 and C8, which are regulated by *luxR/luxI* genes and *ainR/ainS* genes, respectively. Both LuxI/AI-1 and LuxS/AI-2 control the bioluminescence of *A fischeri*. *Bacillus subtilis* is gram-positive bacteria that is distributed in soil and decaying organic matter and is widely used in the detection of pollutant toxicity. *Bacillus subtilis* has the LuxS/AI-2 system.

In the present study, close attention is paid to the toxicities of QSIs to *A fischeri* and *B subtilis* with exposing time going using luminous intensity and mass growth as the bioassay end point, respectively. In addition, the toxic mechanisms on gram-negative bacteria and gram-positive bacteria are also discussed. This study provides theoretical support for environmental risk assessment on QSIs.

## Methods and Materials

All the compounds were purchased in the highest commercially available purity (99%) from Sigma-Aldrich (St. Louis, MO, USA). The information of the compounds is listed in Table 1. Dimethyl sulfoxide at a concentration below 0.1% was used to increase the solubility of the compounds. *Aliivibrio fischeri* (No. ATCC 7744) was obtained from the Institute of Microbiology, Chinese Academy of Sciences (Beijing, China). *Bacillus subtilis* (No. 168) was supplied by Biovector Science Lab, Inc (Beijing, China).

**Table 1.** Name, Abbreviation, CAS, and Structural Formula of the Tested Chemicals.

Category	Name	Abbreviation	CAS	Structure	SL (%)
Furanone	Furaneol acetate	FA	4166-20-5	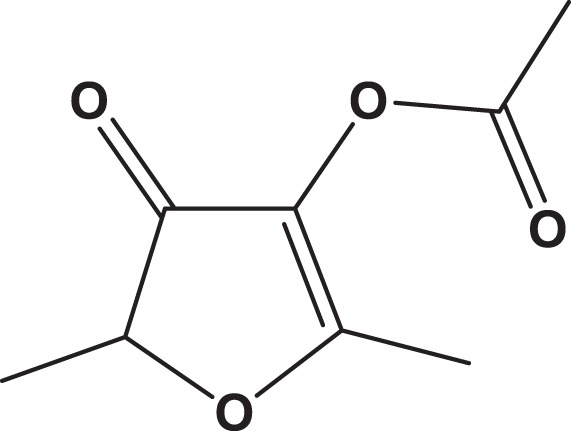	7.3
Pyrrolone	dl-Pyroglutamic acid	2P5CA	149-87-1	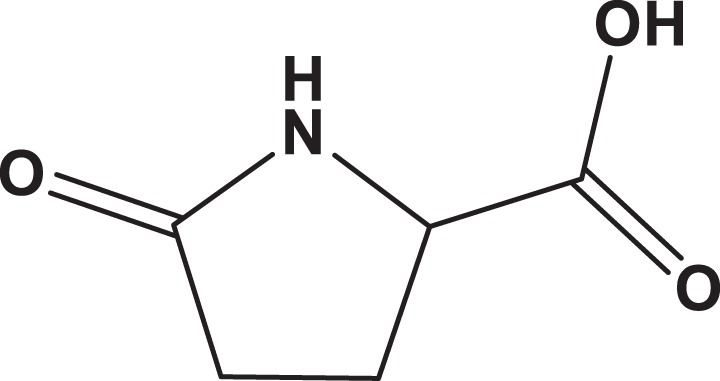	5.6
Pyrrole	l-(+)-Prolinol	S2P	23356-96-9	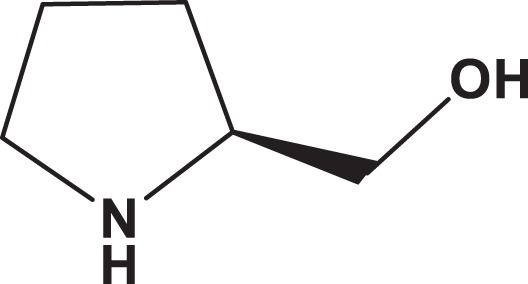	3.0
Pyrrole	d-Prolinol	R2P	68832-13-3	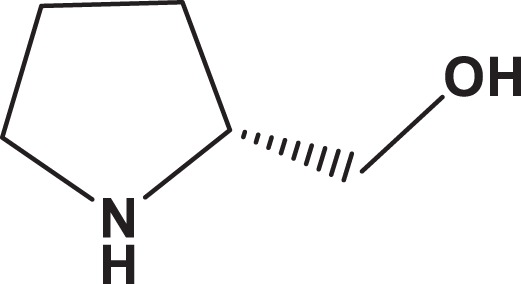	3.2

Abbreviations: CAS, Chemical Abstracts Service; SL, solute loss.

To validate the stability of QSIs, 1 mM QSIs solution were prepared and treated at 37°C for 24 hours. The concentrations were measured by high-performance liquid chromatography (Waters 2695 high-performance liquid chromatograph with a C18 column [Sunfire, 5 μm, 4.6 mm ×150 mm] and ultraviolet/visible detector [Waters 2489], Waters Corporation, Taunton, MA, USA). Methanol and water were used as mobile phase. The concentration ratio was 7:3 and the flow rate was 0.5 mL/min. The solute loss is shown in Table 1.

Prior to the toxicity test, *B subtilis* strains and *A fischeri* strains were separately inoculated in 5-mL Lysogeny broth (LB) and cultivated at 37°C till log growth phase. Then, the 2 bacterial solutions were diluted by 1% (*B subtilis*) and 2% (*A fischeri*) NaCl solution, respectively, to ensure bacterial density about 10^3^ CFU/mL.

As for *A fischeri*, the test compounds were dissolved and diluted into gradient concentrations using 2% NaCl. Then, 80 μL of diluted chemical solution, 80 μL of 2.5-fold LB, and 40 μL of diluted bacterial solution were added into a 96-well plate orderly. The bioluminescence values were measured per hour during a 24-hour culturing using microplate reader (Berthold Technologies Ltd, Bad Wildbad, Germany).

The toxicity test method of *B subtilis* is similar to that of *A fischeri*. The difference is that the concentration of NaCl solution was adjusted from 2% to 1% in the diluting process. Then, the OD_600_ values of *B subtilis* solutions were measured per hour during a 24-hour exposing time using Bioscreen automatic growth curve analyzer (Bioscreen, Helsinki, Finland).

In each experiment, we set wells with no test compound in them as the control group. All the toxicity tests were operated in triplicates. The results are obtained using the following equation:

$$\text{Inhibition}\left(100\text{\%}\right)=\frac{RU_{0}-RU_{\text{i}}}{RU_{0}}\times 100,$$

where *RU*_0_ is the bioluminescence value (*A fischeri*) or OD_600_ (*B subtilis*) of the control groups. *RU*_i_ is the bioluminescence value or OD_600_ of the groups treated by compounds at concentration i. Statistical analyses were performed using ORIGIN 8.1 software (OriginLab Inc, Northampton, MA, USA).

## Results and Discussion

### Assay of Bioluminescence Curve and Bacterial Growth Curve

**Figure 2.**
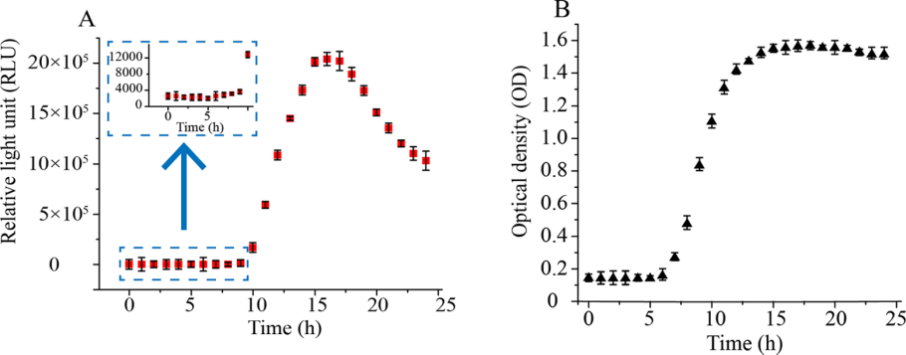
Bioluminescence (HV) of *Aliivibrio fischeri* (A) and biomass (OD) of *Bacillus subtilis* (B) over 24 hours.

The bioluminescence value of *A fischeri* and biomass (OD_600_) of *B subtilis* over 24 hours were determined in the present study. Figure 2 shows the growth curves of *A fischeri* (A) and *B subtilis* (B). The bioluminescence values of *A fischeri* were low at the lag phase between 0 and 8 hours, and rapidly increased to a peak at 14 hours at the log phase (9-14 hours). Then, the bioluminescence values showed a decline after 15 hours (Figure 2A). The changes of bioluminescence were mainly regulated by QS system.^18^ As for *B subtilis*, the bacteria were in the lag phase between 0 and 6 hours and entered into log growth phase at 6 hours, after which the OD_600_ values sharply increases to a peak at 14 hours and kept stable till 24 hours **(** Figure 2B).

### Toxicity Tests for *A Fischeri* over 24 hours

To investigate how exposing time impacts the toxicity of QSIs to bacteria, the toxicities of 4 QSIs to *A fischeri* were determined from 0 to 24 hours. The results revealed the toxic effect of the 4 compounds were similar, and furaneol acetate (FA) is taken, for example, to analyze the rules. Other results are given in Supplementary Figures 1 to 3.

**Figure 3.**
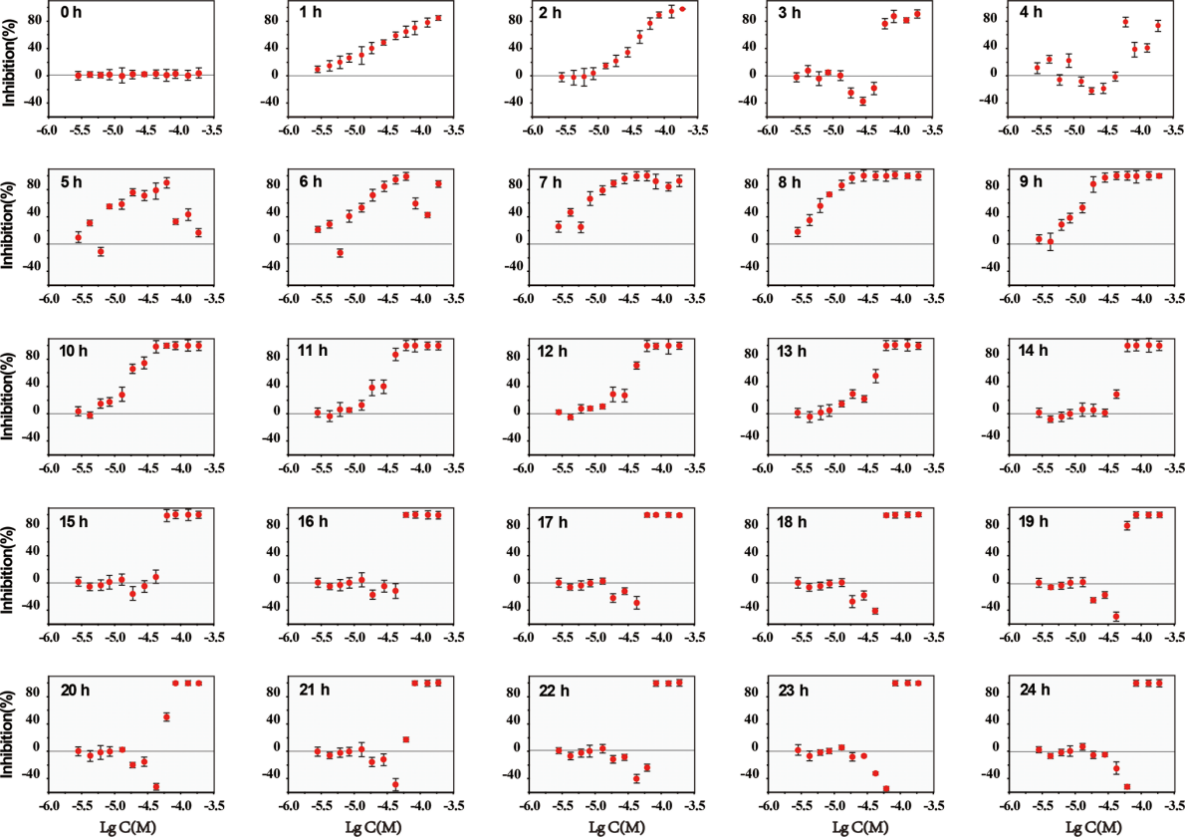
Dose–response relationship between FA and *Aliivibrio fischeri* over 24 hours. Hormesis effect arises from hours 3 to 6 and 15 to 24 (within 24 hours). FA indicates furanone acetate.

The dose–response relationship between FA and bioluminescence of *A fischeri* from hours 0 to 24 is shown in Figure 3. The toxic effect can be divided into 4 stages according to whether or not there is hormesis phenomenon. A detailed analysis of the dose–response relationship is given.

**Figure 4.**
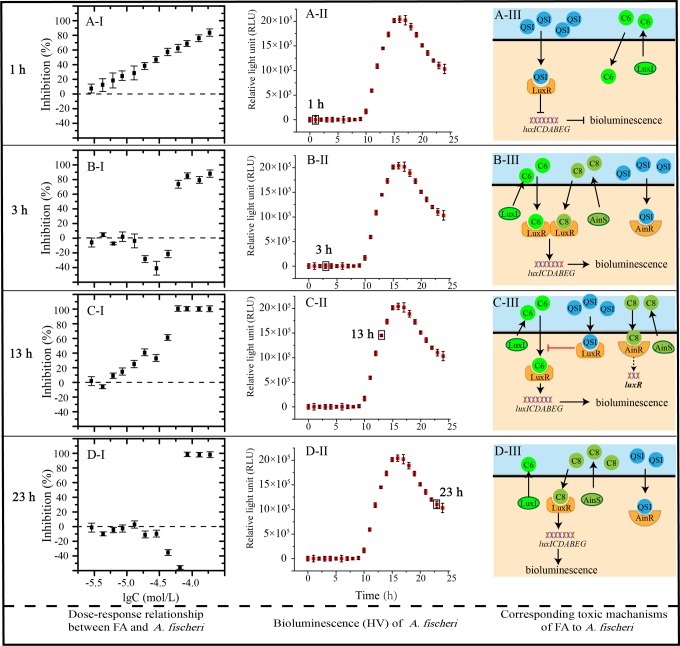
Dose–response relationship at particular points of growth and corresponding mechanisms of toxicity of FA to *Aliivibrio fischeri*. (A), (B), (C), and (D) show the dose–response relationship (I), the growth phase (II), and toxic mechanism (III) of FA to *A fischeri* at hours 1, 3, 13, and 23, respectively. FA indicates furanone acetate.

From hours 1 to 2, FA shows merely inhibition on the bioluminescence of *A fischeri*, and the inhibition is enhanced with concentration increasing (Figure 3). Since FA is similar with N-acyl homoserine lactones (AI-1) on molecular structure, water solubility, and lipid solubility,^19,20^ The FAs can quickly enter the cells and competitively bind to the LuxR protein. This process reduces LuxR-C6 complex, leading to an inhibition on bioluminescence by disrupting the expression of *luxICDABEG* genes (Figure 4A-III).

From hours 3 to 6, FA exerts hormesis effect on *A fischeri* (Figure 3). Hormesis refers to a phenomenon that is characterized by low-dose stimulation and high-dose inhibition. Numerous studies have demonstrated that hormesis is a common phenomenon in the laboratory and natural environment.^21–24^ As shown in Figure 4 B-I, with the concentration (−lgC (M)) of FA increases from −4.82 to −4.35, the inhibiting rate declines to negative. That is to say, FA enhances the bioluminescence. The maximum stimulating rate reaches to 34.69% with FA at the concentration (−lgC (M)) of −4.55. Since bacteria are in lag phase (Figure 4B-II), AinR protein and C8 begin to be expressed abundantly, while the amount of LuxR protein and C6 are relatively low.^25^ Furaneol acetate can more easily bind to AinR protein than C8, leading to an increase in dissociative form of C8.^23^ Next, C6 and C8 bind to LuxR protein, which induces a promotion on bioluminescence of *A fischeri* (Figure 4B-III). This is why FA can stimulate the bioluminescence. However, with the concentration of FA goes up, FA can also bind to LuxR protein. This binding makes bioluminescence weakened, thus the inhibition recovers.

From hours 7 to 14, hormesis effect disappears and only inhibition can be observed. Take the hour 13, for example, as the bacteria enter into log phase **(** Figure 4C-II), LuxR protein, AinR protein, and signal molecules are synthesized greatly.^26^ When exposing to low concentration of FA, LuxR protein is not consumed completely by FA. Therefore, the binding of LuxR protein with C6 results in considerable bioluminescence and the inhibition is limited. However, with FA concentration increase, more FA binds to LuxR protein instead of C6, thereby making the bioluminescence inhibited gradually (Figure 4C-III). With time goes by, low concentration of FA is consumed by the binding with LuxR protein, so C6 can rebind to LuxR and a certain degree of bioluminescence recovers. This is why inhibition is gradually weakened when FA is at low concentration with time increasing from hours 8 to 14.

From hours 15 to 24, hormesis effect occurs again. The maximum promotion reaches to 58.13% at FA concentration (−lgC (M)) of hours 4.21 at hour 23 (Figure 4D-I). As the bacteria enter into the later stage of the stationary phase (Figure 4D-II), there is a strong possibility that C8 production and the expression of AinR remain at a high level. As described previously, FA can readily bind to AinR protein rather than LuxR protein, enabling LuxR protein to form LuxR-C8 complex with C8 (Figure 4D-III). As a result, low concentration of FA stimulates bioluminescence. That is to say, excessive binding of AI-1 and LuxR protein derived from the presence of FA is the cause of bioluminescence facilitation. Nevertheless, with the concentration of FA increases, there remains extra FA to bind to LuxR protein, which depresses the formation of LuxR-C8 complex and subsequent expressions of genes involving bioluminescence. Thus, high concentration of FA exerts inhibition on bioluminescence.

### Toxicity Tests for *B subtilis* Over 24 hours

The toxicities of 4 QSIs to *B subtilis* were determined. The experimental results suggest that 4 QSIs assume similar effect to *B subtilis* with time. Furaneol acetate is taken, for example, to analyze the rules; other results are given in Supplementary Figures 4 to 6.

**Figure 5.**
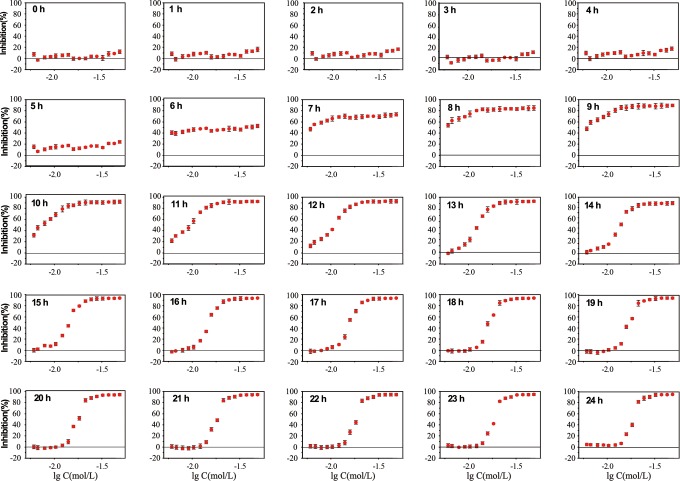
Dose–response relationship between FA and *Bacillus subtilis* over 24 hours. Only inhibitory effect can be observed within 24 hours, toward which the dose–response relationship tends to be an S-shaped curve. FA indicates furanone acetate.

The toxicity of FA to *B subtilis* (0-24 hours) is shown in Figure 5. Toxic effect can be divided into 3 stages: (1) from hours 0 to 5, only slight inhibition is observed (up to 20.0%); (2) from hours 6 to 12, inhibition reaches over 41.2% on each occasion (hours 6-9), then weakens when FA is at low concentration (hours 9-12); and (3) the toxic effect tends to be stable S shape (from hours 13 to 24). Apparently, time-dependent toxicities of FA to *B subtilis* are different from those to *A fischeri*; this is reasonably derived from diverse QS systems.

Previous studies have examined the inhibitory effect of furanone on *B subtilis* ^27,28^ and indicated that furanone produced toxicity by acting on Al-2 QS system of *B subtilis.* ^29^ In Al-2 QS system, LuxS protein produces AI-2 signal molecule to regulate the QS system. However, exogenous FA could covalently bind to LuxS protein, deactivating LuxS and decreasing the production of AI-2 signal molecule; this process yields a block to normal bacterial activities. We combine growth phase and the toxic mechanism to analyze the dose–response relationship between FA and *B subtilis* over 24 hours.

**Figure 6.**
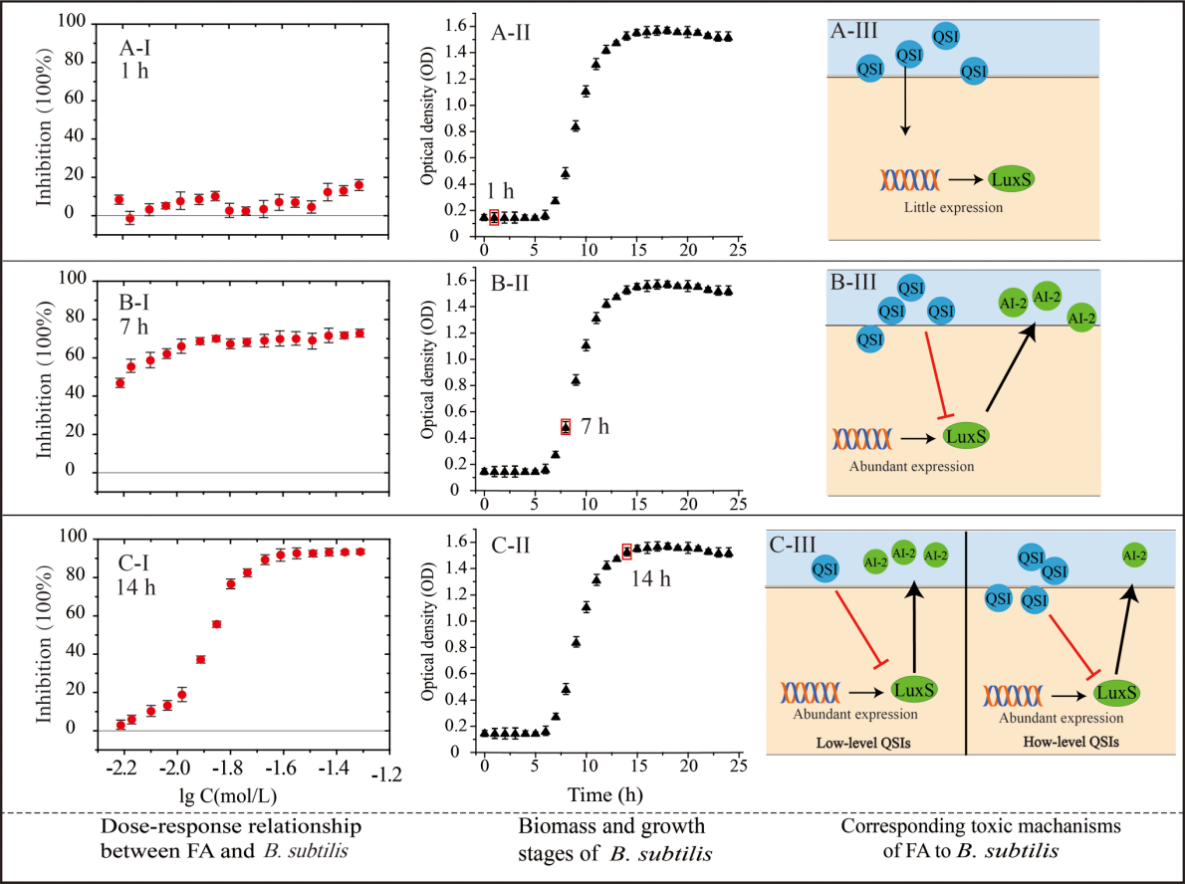
Dose–response relationship at particular points of growth and corresponding mechanisms of toxicity of FA to *Bacillus subtilis*. (A), (B), and (C) show the dose–response relationship (I), the growth phase (II), and toxic mechanism (III) of FA to *B subtilis* at hours 1, 7, and 14, respectively. FA indicates furanone acetate.

From hours 1 to 5, only marginal inhibitions are observed (Figure 6A-I). Since the bacteria are in the lag growth phase (Figure 6A-II), the bacterial density and the expression of LuxS protein are relatively low, as well as the combination between FA and LuxS protein. Therefore, the inhibition of FA on *B subtilis* is limited.

From hours 6 to 12, FA exerts considerable inhibition on *B subtilis*. With time increasing, the inhibiting rate descends when FA is in low concentration region (Figure 5). As *B subtilis* enters into log growth phase at hour 7 (Figure 6B-II), high level of LuxS protein is expressed and FA begins to bind to LuxS protein. It can be inferred that this process decreases the production of AI-2. Since AI-2-mediated LuxR relates to biofilm formation and morphogenetic genes expressions,^30,31^ FA exhibits considerable inhibition (>40%) on growth from hours 6 to 8. However, with time goes by, especially after hour 10, low concentration of FA is consumed away by binding with LuxS protein. The reminding LuxS protein generates AI-2, and the normal physiological activity is maintained. This is why low concentration of FA exerts low toxicity over time.

From hours 13 to 24, the dose–response relationship tends to be S-shaped (Figure 5). As bacteria enter into stagnation phase from hour 14 (Figure 6C-II), the concentration of LuxS protein and AI-2 tends to be stable. When exposing to low-level FA, the generation of AI-2 is slightly impacted, so low level of FA shows low toxicity. However, when exposed to high-level FA, there is sufficient FA to bind to LuxS protein and AI-2 synthesis is blocked. Summing up, the dose–response relationship tends to be S shape from hours 13 to 24.

### Comparison of the Toxic Effect to *A fischeri* and *B subtilis*

Quorum sensing inhibitors generate different toxic effects on *A fischeri* and *B subtilis*. Firstly, dose–response relationships exhibit disparate changing trends. The toxicity of QSIs to *A fischeri* shows 4 stages with exposing time increasing: (I) only inhibitory effect exists, (II) hormesis effect occurs, (III) hormesis effect disappears and overall inhibition emerges, and (IV) hormesis phenomenon occurs again and the stimulation is enhanced with time. The stimulation on bioluminescence described in stages II and IV is ascribed to excessive binding of AI-1 and LuxR protein derived from the presence of QSIs. As for *B subtilis*, the toxic effect of QSIs shows a relatively simple process with 3 stages: (1) no obvious toxic effect exists, (2) an overall inhibition arises but the inhibitions of low-level of QSIs are weakened as time goes, and (3) a stable S-shaped curve forms. The differences in toxic effect result from disparate QS systems. The LuxR/LuxI-type QS system of *A fischeri* is complex with 2 signal molecules C6 and C8, which play roles differently at each bacterial growth phases. However, the QS system regulated by AI-2 signal molecule in *B subtilis* is relatively simple, as well as the toxic dose–response relationship.

Secondly, the toxic level of FA to *A fischeri* and *B subtilis* differs. As for *A fischeri*, no observed effect concentration (NOEC, −lgC (M)) is 4.56, and EC_90_ (lgC (M)) is 4.10 at hour 24. In comparison, NOEC (lgC (M)) and EC_90_ (lgC (M)) of FA to *B subtilis* are 1.91 and 1.58, respectively. It is clear that FA exerts stronger toxicity on *A fischeri* than on *B subtilis.*

## Conclusions

The experimental results show that the toxic level of QSIs to gram-negative bacteria (*A fischeri*) is greater than that to gram-positive bacteria (*B subtilis*). Therefore, in the risk assessment process, these differences should be taken into consideration. Furthermore, the toxic effect of QSIs to gram-negative bacteria shows an intermittent hormesis phenomenon, so the low-dose stimulation should receive more attention when the assessment is conducted.

## Supplemental Material

Supplemental Material, supplementary_data - Time-Dependent Toxicities of Quorum Sensing Inhibitors to *Aliivibrio fischeri* and *Bacillus subtilis* Click here for additional data file. Supplemental Material, supplementary_data for Time-Dependent Toxicities of Quorum Sensing Inhibitors to *Aliivibrio fischeri* and *Bacillus subtilis* by Yueheng Zhang, Jinyuan Song, Ting Wang, Haoyu Sun, Zhifen Lin, and Yinjiang Zhang in Dose-Response
