# Supplementary material for: Time-Dependent Toxicities of Quorum Sensing Inhibitors to Aliivibrio fischeri and Bacillus subtilis
Source: Dose Response. 2019 Feb 25;17(1):1559325818822938. doi: 10.1177/1559325818822938 (PMC6390225; doi:10.1177/1559325818822938)
Supplement: Supplemental Material, supplementary_data - Time-Dependent Toxicities of Quorum Sensing Inhibitors to Aliivibrio fischeri and Bacillus subtilis [file supplementary_data.pdf]

Supplementary data

Supplementary data includes 6 figures

Appendix Fig. 1 Dose–response relationship between 2P5CA and *A. fischeri* over 24 h

Appendix Fig. 2 Dose–response relationship between S2P and *A. fischeri* over 24 h

Appendix Fig. 3 Dose–response relationship between R2P and *A. fischeri* over 24 h

Appendix Fig. 4 Dose–response relationship between 2P5CA and *B. subtilis* over 24 h

Appendix Fig. 5 Dose–response relationship between S2P and *B. subtilis* over 24 h

Appendix Fig. 6 Dose–response relationship between FA and *B. subtilis* over 24 h

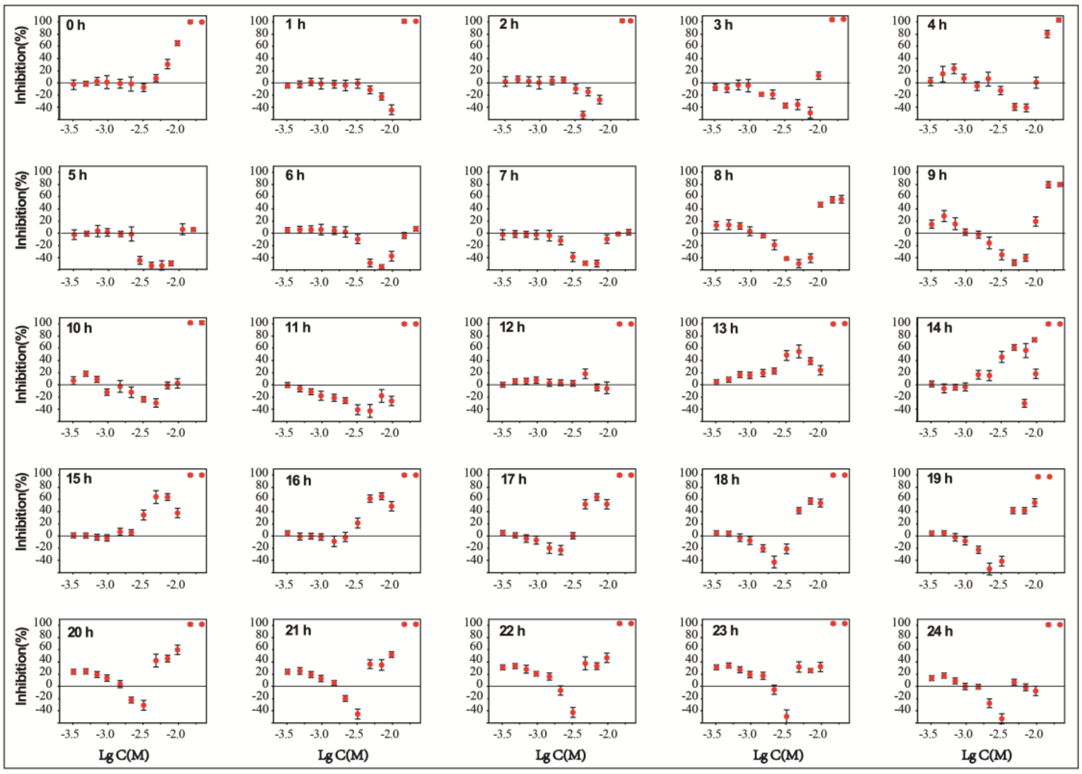

Appendix Fig. 1 Dose–response relationship between 2P5CA and *A. fischeri* over 24 h

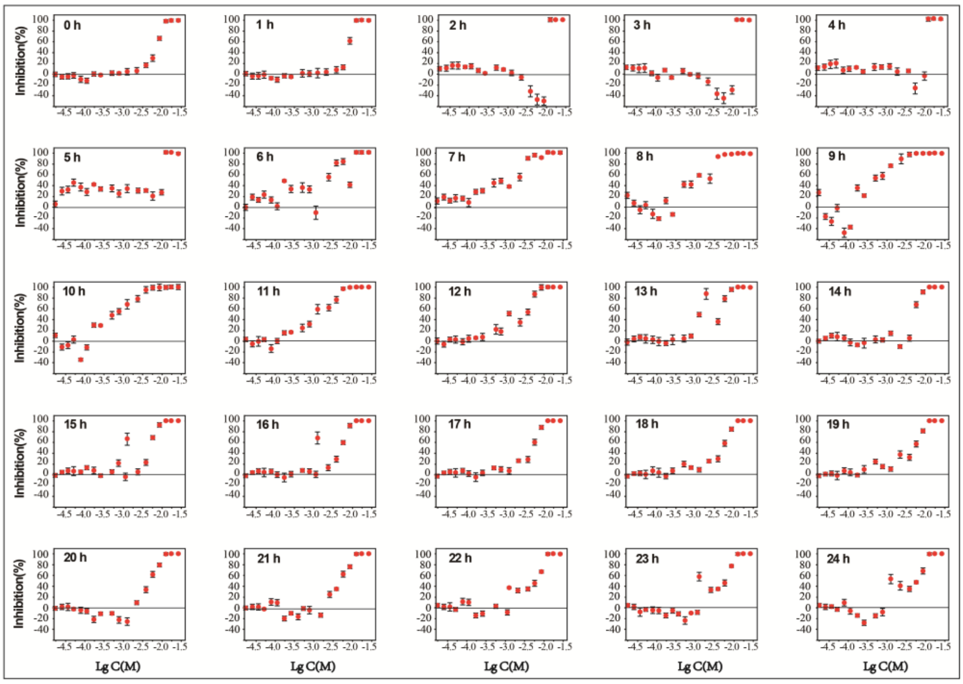

Appendix Fig. 2 Dose–response relationship between S2P and *A. fischeri* over 24 h

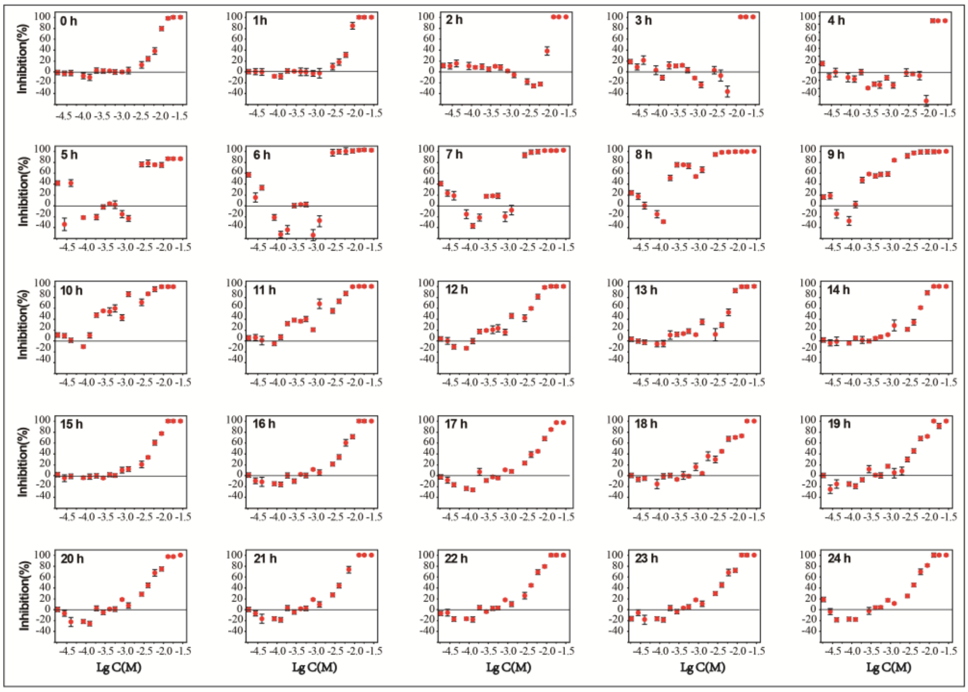

Appendix Fig. 3 Dose–response relationship between R2P and *A. fischeri* over 24 h

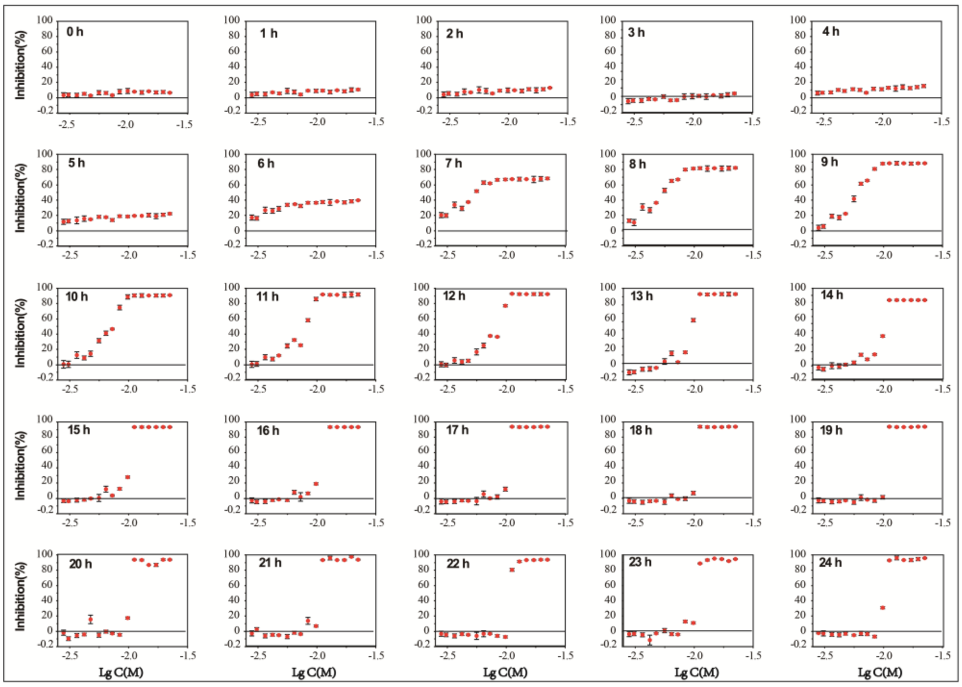

Appendix Fig. 4 Dose–response relationship between 2P5CA and *B. subtilis* over 24 h

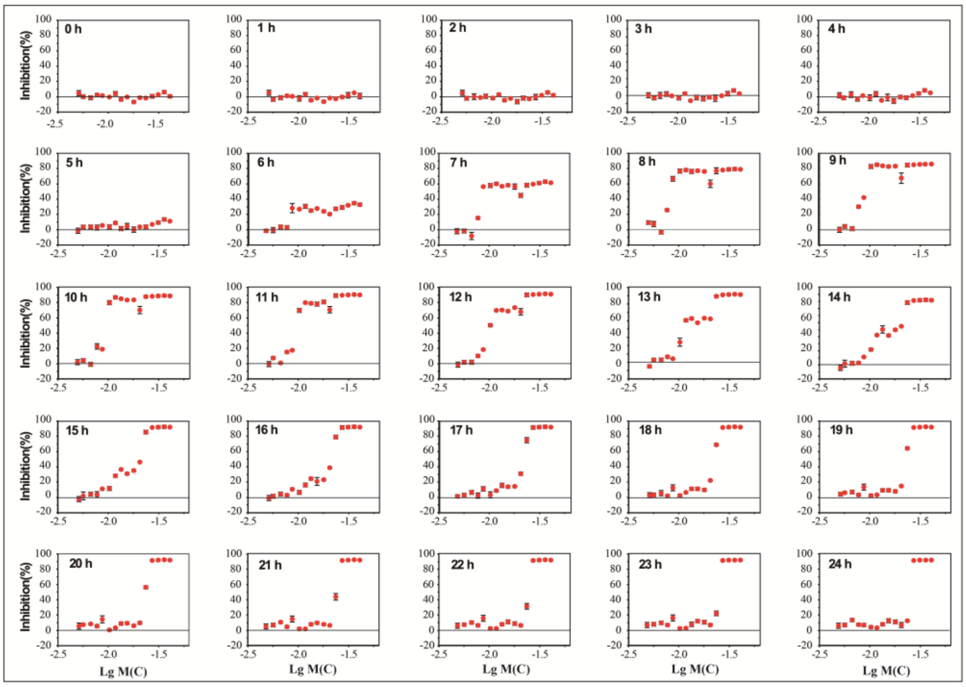

Appendix Fig. 5 Dose–response relationship between S2P and *B. subtilis* over 24 h

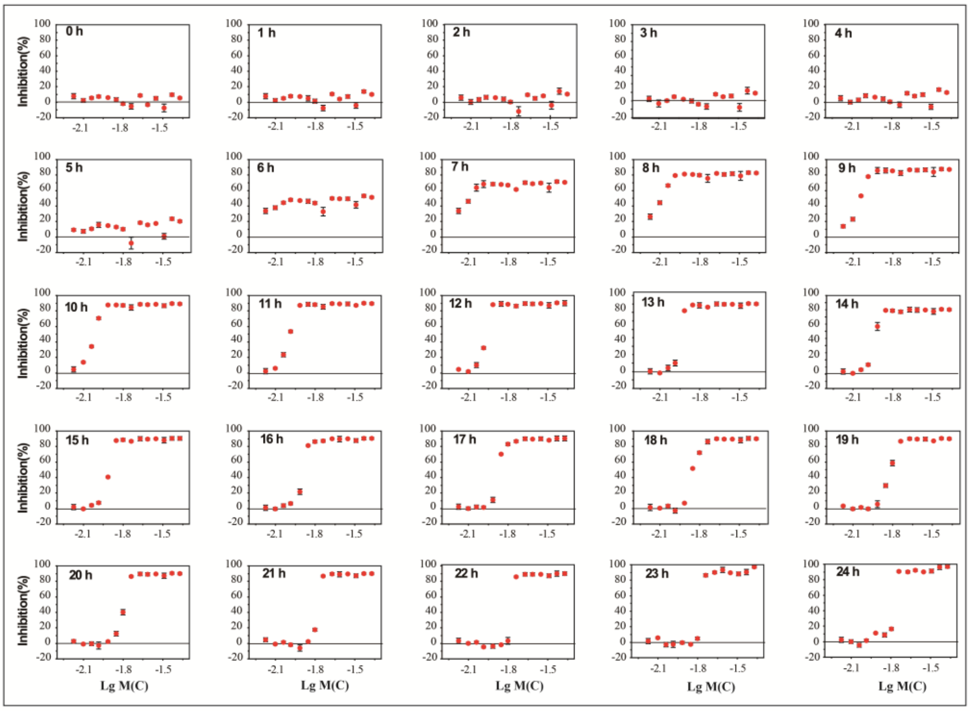

Appendix Fig. 6 Dose–response relationship between R2P and *B. subtilis* over 24 h
